# Evaluation of Non-Laboratory and Laboratory Prediction Models for Current and Future Diabetes Mellitus: A Cross-Sectional and Retrospective Cohort Study

**DOI:** 10.1371/journal.pone.0156155

**Published:** 2016-05-23

**Authors:** Chang Ho Ahn, Ji Won Yoon, Seokyung Hahn, Min Kyong Moon, Kyong Soo Park, Young Min Cho

**Affiliations:** 1 Department of Internal Medicine, Seoul National University College of Medicine, Seoul, Korea; 2 Department of Internal Medicine, Healthcare System Gangnam Center, Seoul National University Hospital, Seoul, Korea; 3 Medical Research Collaborating Center, Seoul National University Hospital, Seoul, Korea; 4 Department of Internal Medicine, Boramae Medical Center, Seoul National University College of Medicine, Seoul, Korea; College of Medicine, National Taiwan University, TAIWAN

## Abstract

**Background:**

Various diabetes risk scores composed of non-laboratory parameters have been developed, but only a few studies performed cross-validation of these scores and a comparison with laboratory parameters. We evaluated the performance of diabetes risk scores composed of non-laboratory parameters, including a recently published Korean risk score (KRS), and compared them with laboratory parameters.

**Methods:**

The data of 26,675 individuals who visited the Seoul National University Hospital Healthcare System Gangnam Center for a health screening program were reviewed for cross-sectional validation. The data of 3,029 individuals with a mean of 6.2 years of follow-up were reviewed for longitudinal validation. The KRS and 16 other risk scores were evaluated and compared with a laboratory prediction model developed by logistic regression analysis.

**Results:**

For the screening of undiagnosed diabetes, the KRS exhibited a sensitivity of 81%, a specificity of 58%, and an area under the receiver operating characteristic curve (AROC) of 0.754. Other scores showed AROCs that ranged from 0.697 to 0.782. For the prediction of future diabetes, the KRS exhibited a sensitivity of 74%, a specificity of 54%, and an AROC of 0.696. Other scores had AROCs ranging from 0.630 to 0.721. The laboratory prediction model composed of fasting plasma glucose and hemoglobin A1c levels showed a significantly higher AROC (0.838, *P* < 0.001) than the KRS. The addition of the KRS to the laboratory prediction model increased the AROC (0.849, *P* = 0.016) without a significant improvement in the risk classification (net reclassification index: 4.6%, *P* = 0.264).

**Conclusions:**

The non-laboratory risk scores, including KRS, are useful to estimate the risk of undiagnosed diabetes but are inferior to the laboratory parameters for predicting future diabetes.

## Introduction

The prevalence of diabetes mellitus is increasing worldwide. The International Federation of Diabetes estimates that 382 million people globally have diabetes as of 2013, and this number will increase by 55% until 2035 [1]. The pathogenesis of diabetes mellitus is very complex, which encompasses pancreatic beta-cell dysfunction, decreased glucose uptake by skeletal muscle, increased hepatic glucose production, increased lipolysis, chronic low-grade inflammation, and so on [2, 3]. Although tremendous efforts have been made to find the cure for diabetes [4], no successful treatment to reverse diabetes exists as of yet. Therefore, prevention is the most important strategy to solve the global epidemic of diabetes. Several large randomized controlled trials demonstrated that early interventions in the form of lifestyle modifications or medications can delay or prevent type 2 diabetes [5, 6]. Therefore, identifying individuals who are undiagnosed or who are at high risk of developing diabetes is of paramount importance to fight the global epidemic of diabetes. However, a significant proportion of diabetes patients are unaware of their condition and leave it untreated. It is estimated that 27.8% of diabetes patients in the United States [7] and 27.3% in Korea are undiagnosed [8]. An effective screening program is essential to identify this population with potentially preventable health outcomes.

Various risk scores have been developed for diabetes screening [9–11]. Each risk score was designed to identify undiagnosed diabetes, incident diabetes, or both. Scores were developed based on various populations with distinct ethnic and clinical backgrounds [9–11]. Established risk factors for diabetes, such as central obesity, a family history of diabetes, and old age were included in the majority of the risk scores [10]. However, other potential risk factors, such as steroid use, the intake of red meat, and alcohol consumption were only included in a subset of the risk scores [10]. The differences in the primary end points of the risk scores, the population that the risk score was derived from, and the risk predictors included in the risk score led to considerable heterogeneity between the risk scores. The risk scores should be used with caution before applying them to different populations.

The risk scores also vary if they are solely based on non-laboratory parameters or include laboratory parameters [9]. The two types of risk scores, those based on only non-laboratory parameters and those based on both non-laboratory and laboratory parameters, require different amounts of resources and have different ranges of applicability. The scores with non-laboratory parameters are inexpensive and ready to use by a layperson. Therefore, they are more suitable for whole population–based screening. The scores using laboratory parameters generally have better discrimination than the non-laboratory scores, but the additional cost and time requirements limit their use in population-based screening [12]. To justify the use of laboratory parameters in diabetes risk prediction, risk prediction improvement using laboratory parameters needs to be thoroughly evaluated.

Recently, a Korean diabetes risk score composed of non-laboratory parameters was developed based on the results from the Korean National Health and Nutrition Examination Survey (KNHANES) [13]. The Korean Risk Score (KRS) includes age, family history of diabetes and hypertension, waist circumference, smoking status, and alcohol intake as the risk factors for undiagnosed diabetes [13]. Although the KRS was validated in the national survey data, it has not been validated in other population data or evaluated for use in the prediction of incident diabetes. To assess the performance of the KRS and compare it with other previously published risk scores, we performed a comprehensive validation of the KRS in a large independent cohort and compared it with various other risk scores for both undiagnosed and incident diabetes. Further, we evaluated the improvement of the risk prediction model after incorporating laboratory parameters into the KRS.

## Methods

### Subjects and study design

Subjects were recruited at Seoul National University Hospital Healthcare System Gangnam Center. The participants in the health screening program were asked to provide their results for clinical research, and the database was constructed after the encryption of personal information. The written informed consent was obtained for all study participants. This study was approved by the Institutional Review Board of Seoul National University Hospital (IRB No. 1308-004-507) and conducted according to the Declaration of Helsinki.

#### Cross-sectional validation for the screening of undiagnosed diabetes

Among the individuals who visited the healthcare center from January 1, 2011, to September 31, 2012, a total 28,857 individuals completed the health screening program, including measurements of fasting plasma glucose (FPG) and hemoglobin A1c (HbA1c) levels. We excluded 75 individuals under the age of 20, 564 individuals with missing data on waist circumference or body weight, and 1,543 individuals who reported having known diabetes. Consequently, 26,675 individuals formed the study population for the cross-sectional validation. In cases of individuals who had multiple visits during the study period, only the data from the first visit were used.

#### Longitudinal validation for the prediction of incident diabetes

For the longitudinal validation, we constructed a retrospective cohort with a baseline visit during the period from February 1, 2004, to December 31, 2005, and a follow-up visit during the period from January 1, 2011, to September 31, 2012. Totally, 4,147 individuals had both baseline and follow-up visits and completed the health screening program. From this eligible population, 75 individuals under the age of 20, 13 individuals with missing data on waist circumference or body weight, and 781 individuals with incomplete follow-up data were excluded. The 144 individuals with previously diagnosed diabetes and 105 individuals with newly diagnosed diabetes at the baseline visit were also excluded. As a result, 3,029 individuals formed the study population for the longitudinal validation, with a mean follow-up duration of 6.2 years.

### Clinical and laboratory evaluation

A questionnaire was used to gather information on medical history, family history, health-related habits, and physical activity. The medical history included current medications and any previous diagnosis of diabetes, hypertension, or dyslipidemia. Individuals who reported having hypertension or were taking antihypertensive medication or whose blood pressure was ≥140/90 mmHg were defined as having hypertension. A family history of diabetes was confined to first-degree relatives. Smoking status was classified as current smokers, ex-smokers (not currently smoking but had smoked at least 5 packs of cigarettes in their lifetime), and never-smokers. Alcohol consumption was calculated as an average daily number of drinks based on the frequency of drinking per week and the amount of alcoholic beverage consumed. Subjects were classified as physically active if they performed regular exercise more than once per week. The blood samples were collected after a 12-hour overnight fast. Plasma glucose, HbA1c, total cholesterol, HDL cholesterol, and triglyceride levels were measured. The plasma glucose level (intra-assay and inter-assay coefficient of variation [CV] <2.0%) was determined with the hexokinase method using an Architect ci8200 analyzer (Abbott Laboratories, Abbott Park, IL, USA). Total cholesterol, HDL cholesterol, and triglyceride levels (intra-assay and inter-assay CV <1.5% for total cholesterol; <2.5% for HDL cholesterol; <1.5% for triglyceride) were determined with enzymatic kits using the Architect ci8200 analyzer. HbA1c levels (intra-assay and inter-assay CV <2.0%) were determined with an immunoturbidimetric assay using Cobas Integra 400 (Roche Diagnostics GmbH, Mannheim, Germany).

### Definition of diabetes mellitus

The subjects who answered “yes” to the question “Have you ever been diagnosed with diabetes by a physician?” or who were taking antidiabetic medications were defined as having “known diabetes.” The subjects who were first diagnosed with type 2 diabetes based on the result of the fasting blood test at the health screening program were classified as “undiagnosed diabetes.” Among the subjects who did not have diabetes at the baseline visit, the subjects who were diagnosed with diabetes or had undiagnosed diabetes at the follow-up visit were classified as “incident diabetes.” Diabetes was diagnosed in subjects with FPG ≥ 126 mg/dl or HbA1c ≥ 6.5% according to the 2010 revision of the diagnostic criteria of diabetes by American Diabetes Association (ADA) [14].

### Risk scores

We adopted risk scores from 3 recently published systematic reviews on risk prediction models for type 2 diabetes [9–11]. According to the standard methodology for systematic reviews, each systematic review searched published articles that reported risk prediction models for type 2 diabetes. The timings of the searches in the 3 reviews were January 2011 [11], February 2011 [9], and May 2011 [10]. We further searched other studies reporting risk scores published from January 2011 to May 2013 on PubMed and Google Scholar using the following search string: ((“diabetes” OR “type 2 diabetes”) AND (“score” OR “model” OR “prediction”)). Among the identified risk scores, the scores using only non-laboratory parameters were included. A total of 16 risk scores were included in the comparative analysis with the KRS (S1 Table). When the definition of each variable was not identical with our study, we tried to use the best available variable. Two variables, current use of corticosteroid and history of gestational diabetes, were omitted because those conditions were not evaluated in our study.

### Statistical analysis

We classified subjects according to diabetes status for descriptive statistics. Continuous variables are expressed as the means ± SD, and categorical variables are presented as percentages. The difference between each group was analyzed by the one-way ANOVA with Tukey’s post hoc test or *t* test for continuous variables and a chi-square test for categorical variables. To validate and compare various risk scores, we calculated the proportion of high-risk individuals, sensitivity, specificity, positive predictive value, negative predictive value, and Youden index (sensitivity + specificity -1) for each model. The area under the receiver operating characteristic curve (AROC) was also calculated as a discrimination index. In the study population for cross-sectional validation, we applied risk prediction models to detect undiagnosed diabetes. In the study population for longitudinal validation, we calculated risk scores based on the values at baseline visit to predict incident diabetes at a follow-up visit.

To compare the performance of the non-laboratory risk score and laboratory parameters for the prediction of incident diabetes, we developed risk prediction models composed of laboratory parameters in the study population for longitudinal validation with multivariate logistic regression analysis. Laboratory parameters were selected in the multivariate logistic regression analysis with a backward stepwise methods (the cutoff of *P* values was <0.05). Aforementioned measures including AROC were calculated for these risk prediction models of laboratory parameters. The net reclassification index (NRI) and integrated discrimination improvement (IDI) [15] of each parameter and the combined laboratory parameters were also calculated in comparison with the KRS. Additionally, to assess the effectiveness of the risk scores as a diabetes screening program, we simulated the application of the KRS and combined risk prediction model of the KRS and laboratory parameters (CRPM) to the baseline data of the longitudinal study with a total of 3,134 individuals without known diabetes at the baseline visit, which were 3,029 normal individuals plus 105 undiagnosed diabetes at baseline. A *P* value <0.05 was considered to be statistically significant. All statistical analyses were performed using SPSS v18.0 (SPSS Inc. Chicago, IL, USA) or R v3.0.1 (R Foundation for Statistical Computing, Vienna, Austria).

## Results

### Characteristics of the study populations

The characteristics of the study population for the cross-sectional validation according to diabetes status are summarized in Table 1. The prevalence of the diabetes was 8.4%, including undiagnosed diabetes (2.9%). As expected, known risk factors of diabetes, including old age, higher BMI, waist circumference, hypertension, a family history of diabetes, and smoking, were associated with diabetes. The modifiable risk factors of diabetes (obesity, physical inactivity, and alcohol intake) were less prevalent in known diabetes than undiagnosed diabetes. Known diabetes patients had lower BMI (24.6 ± 3.0 vs. 25.6 ± 3.2 kg/m^2^, *P* < 0.001), performed more regular exercise (81.3% vs. 69.7%, *P* < 0.001), and had less alcohol intake (1.6 ± 1.9 vs. 1.9 ± 2.1 glasses/day, *P* = 0.014) than those with undiagnosed diabetes.

**Table 1:** Baseline characteristics of the study population.

	Cross-sectional validation							Longitudinal validation		
Characteristics	Normal (n = 25,859)	Undiagnosed DM (n = 816)	Known DM (n = 1,543)	*P* value (group comparison)*	*P* value (normal vs. undiagnosed DM)†	*P* value (normal vs. known DM)†	*P* value (undiagnosed DM vs. known DM)†	Normal at f/u (n = 2,893)	Incident DM (n = 136)	*P* value
Age (years)	48.5 ± 11.1	55.6 ± 9.5	58.1 ± 9.8	<0.001	<0.001	<0.001	<0.001	47.1 ± 9.8	52.0 ± 8.2	<0.001
Male	13,890 (53.7)	592 (72.5)	1,172 (76)	<0.001	<0.001	<0.001	0.070	1659 (57.3)	95 (69.9)	0.004
Height (cm)	165.7 ± 8.2	166.9 ± 8.3	166.4 ± 7.8	<0.001	<0.001	0.002	0.462	165.7 ± 8.0	166.8 ± 8.0	0.108
Weight (kg)	63.9 ± 11.9	71.5 ± 11.8	68.4 ± 11.1	<0.001	<0.001	<0.001	<0.001	64.2 ± 11.1	70.6 ± 11.3	<0.001
BMI (kg/m^2^)	23.1 ± 3.1	25.6 ± 3.2	24.6 ± 3.0	<0.001	<0.001	<0.001	<0.001	23.3 ± 2.8	25.3 ± 2.9	<0.001
Waist circumference (cm)	83.4 ± 10.3	90.6 ± 8.1	88.6 ± 8.2	<0.001	<0.001	<0.001	<0.001	84.0 ± 7.7	89.3 ± 7.0	<0.001
Hypertension	5,705 (22.1)	407 (49.9)	841 (54.5)	<0.001	<0.001	<0.001	0.032	743 (25.7)	56 (41.2)	<0.001
Family history of diabetes	5,015 (19.4)	258 (31.6)	649 (42.1)	<0.001	<0.001	<0.001	<0.001	610 (21.1)	44 (32.4)	0.002
Current Smoking	4,330 (16.7)	172 (21.1)	323 (20.9)	<0.001	0.001	<0.001	0.934	528 (18.3)	32 (23.5)	0.121
Regular exercise	19,991 (77.3)	569 (69.7)	1,254 (81.3)	<0.001	<0.001	<0.001	<0.001	1767 (61.1)	88 (64.7)	0.396
Alcohol intake (glasses/day)	1.3 ± 1.8	1.9 ± 2.1	1.6 ± 1.9	<0.001	<0.001	<0.001	0.018	1.1 ± 1.9	1.4 ± 2.0	0.079
Fasting plasma glucose (mg/dL)	93.5 ± 9.8	131.3 ± 25.9	132.4 ± 35.3	<0.001	<0.001	<0.001	0.141	96.1 ± 9.8	106.8 ± 10.7	<0.001
HbA1c (%)	5.6 ± 0.3	6.8 ± 0.9	6.9 ± 1.1	<0.001	<0.001	<0.001	<0.001	5.5 ± 0.3	5.9 ± 0.3	<0.001
Total cholesterol (mg/dL)	194.1 ± 33.1	201 ± 40.4	175.9 ± 36.2	<0.001	<0.001	<0.001	<0.001	197.1 ± 32.8	206.0 ± 32.6	0.002
Triglyceride (mg/dL)	107.6 ± 73.8	157 ± 96.9	130.4 ± 90.6	<0.001	<0.001	<0.001	<0.001	108.6 ± 69.4	144.5 ± 80.7	<0.001
HDL cholesterol (mg/dL)	53 ± 11.6	48.2 ± 9.9	48.3 ± 10.5	<0.001	<0.001	<0.001	0.986	54.7 ± 13.2	50.1 ± 13.3	<0.001

Abbreviations: DM, diabetes mellitus; BMI, body mass index; HbA1c, hemoglobin A1c.

Data are the mean ± SD or n (%).

*P values for the group difference were calculated by one-way ANOVA test for continuous variables and chi-square test for categorical variables.

†*P* values for the comparison of each pair of groups were calculated by the post hoc test of ANOVA with Tukey’s method for continuous variables and chi-square tests for categorical variables.

The baseline characteristics of the study population for the longitudinal validation according to diabetes status are summarized in Table 1. Of the subjects who were normoglycemic at baseline, 4.5% developed incident diabetes between the baseline and follow-up visits. The minimum, maximum, and mean durations of follow-up were 5.0, 7.9, and 6.2 years, respectively. Among the various parameters, old age, high BMI, waist circumference, hypertension, and a family history of diabetes were associated with incident diabetes. The proportion of current smokers and the amount of daily alcohol intake were not significantly different between those without diabetes and those with incident diabetes at follow-up.

### Cross-sectional validation for screening of undiagnosed diabetes

We evaluated the performance of different risk scores for the screening of undiagnosed diabetes according to each model’s original cutoff value and new cutoff value (the cutoff value showing the highest Youden index) (Table 2). The KRS demonstrated an AROC of 0.754 (95% CI: 0.740–0.769), a sensitivity of 91%, and a specificity of 40% with the original cutoff value. The sensitivity and specificity of the KRS were 81% and 58%, respectively, with the new cutoff value. The other 16 risk scores exhibited AROCs ranging from 0.697 to 0.782. The 15 models, including the KRS, needed a readjustment of the cutoff values for our study population. With these new cutoff values, the sensitivities of the scores varied from 68% to 85% and the specificities from 42% to 72%. The scores classified an average 37.3% (minimum 29%, maximum 59%) of the subjects as being at high risk for having undiagnosed diabetes. When we analyzed men and women separately, the AROCs of risk scores were higher in women than in men in the cross-sectional validation (S2 Table).

**Table 2:** Performance of risk scores in cross-sectional validation for the screening of undiagnosed diabetes.

Risk score	Cutoff	Patients at high risk (%)	AROC (95% CI)	Sensitivity (%)	Specificity (%)	Youden Index	PPV (%)	NPV (%)	*P* value*
Korean Risk Score (KRS) [13]	≥6	43	0.754 (0.740–0.769)	81	58	40	5.8	99	N/A
-	≥5	61	-	91	40	32	4.6	99	N/A
Australian score (AUSDRISK study) [16]	≥13	29	0.782 (0.769–0.796)	70	72	43	7.4	99	<0.001
-	≥12	35	-	75	66	41	6.6	99	-
Danish score [17]	≥25	36	0.777 (0.763–0.792)	77	65	43	6.6	99	0.001
-	≥31	20	-	55	81	36	8.4	98	-
ADA questionnaire [18]	≥3	36	0.776 (0.762–0.790)	78	65	44	6.6	99	0.004
-	≥5	6	-	21	95	16	11.5	97	-
Japanese score (TOPICS-10 study) [19]	≥8†	32	0.774 (0.760–0.788)	71	70	41	6.9	99	0.002
The Leicester Risk Assessment score [20]	≥17	30	0.773 (0.759–0.788)	71	71	42	7.2	99	0.007
-	≥16	38	-	78	64	42	6.3	99	-
Thai score [21]	≥8	34	0.763 (0.749–0.778)	72	68	39	6.5	99	0.114
-	≥7	46	-	83	55	38	5.5	99	-
Finnish score (DETECT-2 study) [22]	≥5	38	0.759 (0.744–0.774)	75	63	38	6.0	99	0.491
-	≥7	19	-	50	82	32	8.2	98	-
Brazilian score [23]	≥11	46	0.751 (0.737–0.766)	85	55	40	5.7	99	0.723
-	≥18	16	-	42	85	27	8.1	98	-
Indian score [24]	≥18	35	0.751 (0.736–0.766)	74	66	40	6.5	99	0.617
-	≥17	39	-	77	63	39	6.1	99	-
Japanese score (Doi et al.) [25]	≥12	32	0.750 (0.734–0.767)	70	69	39	6.6	99	0.552
-	≥14	23	-	56	79	35	7.7	98	-
Chinese score [26]	≥16	40	0.733 (0.717–0.748)	72	61	33	5.5	99	0.004
-	≥14	62	-	92	39	32	4.6	99	-
British score [12]	≥4	39	0.730 (0.714–0.747)	74	62	36	5.8	99	0.002
-	≥6	15	-	41	86	27	8.5	98	-
Rotterdam model [27]	≥33.9	35	0.727 (0.710–0.743)	68	67	35	6.0	99	0.001
-	≥37	23	-	52	78	30	6.9	98	-
Oman score [28]	≥11	33	0.726 (0.710–0.742)	68	68	35	6.2	99	<0.001
-	≥10	36	-	70	65	35	5.9	99	-
French score (DESIR study) [29]	≥2†	59	0.705 (0.688–0.721)	88	42	29	4.5	99	<0.001
Kuwait score [30]	≥19	37	0.697 (0.681–0.714)	69	64	32	5.6	98	<0.001
-	≥32	7	-	23	93	16	9.8	97	-

Abbreviations: AROC, area under the curve of receiver operating characteristic curve; CI, confidence interval; PPV, positive predictive value; NPV, negative predictive value; N/A, not applicable

Risk scores other than the KRS were arranged in the order of higher AROC values. The performance of different risk scores for screening for undiagnosed diabetes was evaluated with each score’s original cutoff value and the new cutoff value showing the highest Youden index. The results in the upper row of each score are based on the new cutoff value with the highest Youden index. The results in the bottom row of each score are based on the original cutoff value.

*P values for the comparison of ROC curves between the KRS and other scores were calculated using DeLong’s method [31].

†The new cutoff and original cutoff were the same for Japanese scores and French scores.

### Longitudinal validation for prediction of incident diabetes

The comparison of the performance of the different risk scores for the prediction of incident diabetes is summarized in Table 3. All of the risk scores demonstrated lower values of AROCs in the longitudinal validation compare to the cross-sectional validation. The KRS exhibited an AROC of 0.696 (95% CI: 0.656–0.736). The sensitivity and specificity of the KRS were 89% and 37%, respectively, with the original cutoff value, and 74% and 54%, respectively, with the new cutoff value. The other 16 models exhibited AROCs that ranged from 0.630 to 0.721. Fifteen out of 17 models required a readjustment of the cutoff values. With the new cutoff values, the models had sensitivities ranging from 51% to 86% and specificities ranging from 45% to 68%. On average, 42.5% (minimum: 26%, maximum: 57%) of the subjects were classified as being at high risk of developing incident diabetes by risk scores. In addition, the AROC of risk scores were higher in women than in men in the longitudinal validation (S2 Table).

**Table 3:** Performance of risk scores in longitudinal validation for the prediction of incident diabetes.

Risk score	Cutoff	Patients at high risk(%)	AROC (95% CI)	Sensitivity (%)	Specificity (%)	Youden Index	PPV (%)	NPV (%)	*P* value*
Korean Risk Score (KRS) [13]	≥6	47	0.696 (0.656–0.736)	74	54	29	7.1	98	N/A
-	≥5	64	-	89	37	26	6.2	99	-
Australian score (AUSDRISK study) [16]	≥11	43	0.721 (0.679–0.762)	73	58	31	7.6	98	0.121
-	≥12	35	-	62	66	28	7.8	97	-
Finnish score (DETECT-2 study) [22]	≥4	55	0.718 (0.678–0.758)	85	46	31	6.9	99	0.205
-	≥7	21	-	46	80	26	9.8	97	-
Thai score [21]	≥6	56	0.713 (0.675–0.752)	86	45	31	6.9	99	0.309
-	≥7	47	-	76	54	30	7.2	98	-
Danish score [17]	≥25	38	0.700 (0.658–0.742)	65	64	29	7.8	98	0.845
-	≥31	22	-	43	79	22	8.8	97	-
The Leicester Risk Assessment score [20]	≥13	45	0.697 (0.655–0.739)	74	57	30	7.4	98	0.967
-	≥16	35	-	62	66	28	8.0	97	-
Japanese score (TOPICS-10 study) [19]	≥8†	33	0.696 (0.655–0.738)	58	68	26	7.9	97	0.976
Chinese score [26]	≥16	37	0.692 (0.651–0.732)	66	64	31	8.0	98	0.838
-	≥14	58	-	84	43	27	6.4	98	-
Indian score [24]	≥18	38	0.689 (0.648–0.729)	64	63	27	7.5	97	0.703
-	≥17	42	-	68	59	26	7.2	97	-
Japanese score (Doi et al.) [25]	≥12	39	0.688 (0.643–0.733)	69	62	31	7.9	98	0.658
-	≥14	28	-	57	73	30	9.0	97	-
ADA questionnaire [18]	≥3	38	0.688 (0.644–0.731)	66	63	29	7.8	98	0.697
-	≥5	5	-	17	95	12	14.7	96	-
Brazilian score [23]	≥12	40	0.683 (0.640–0.727)	68	61	29	7.5	98	0.554
-	≥18	15	-	30	86	16	9.1	96	-
Oman score [28]	≥9	57	0.680 (0.639–0.722)	82	45	27	6.5	98	0.479
-	≥10	39	-	64	62	26	7.3	97	-
British score [12]	≥4	44	0.670 (0.627–0.714)	69	58	27	7.1	98	0.187
-	≥6	16	-	32	85	17	9.0	96	-
French score (DESIR study) [29]	≥3†	26	0.654 (0.608–0.699)	51	75	27	8.9	97	0.006
Rotterdam model [27]	≥32.9	47	0.646 (0.599–0.693)	71	54	25	6.8	98	0.035
-	≥37	32	-	50	69	19	7.1	97	-
Kuwait score [30]	≥22	40	0.630 (0.587–0.674)	59	61	20	6.6	97	0.002
-	≥32	8	-	18	92	11	10.0	96	-

Abbreviations: AROC, area under the curve of receiver operating characteristic curve; CI, confidence interval; PPV, positive predictive value; NPV, negative predictive value; N/A, not applicable

Risk scores other than the KRS were arranged in order of the higher AROC value. The performance of different risk scores for the prediction of incident diabetes was evaluated with each model’s original cutoff value and new cutoff value showing the highest Youden index. The results in the upper row of each score are based on the new cutoff value with the highest Youden index. The results in the bottom row of each score are based on the original cutoff value.

*P values for the comparison of ROC curves between the KRS and other scores were calculated using DeLong’s methods [31].

†The new cutoff and original cutoff were the same for Japanese scores and French scores.

### Comparison between the laboratory parameters and the Korean risk score

We performed univariate and multivariate logistic regression analysis with the laboratory parameters (FPG, HbA1c, total cholesterol, HDL cholesterol, triglyceride) for the prediction of incident diabetes (S3 Table). All of the laboratory parameters showed a significant association with incident diabetes in the univariate analysis. In the multivariate analysis, only FPG and HbA1c were significant predictors of incident diabetes. The estimated AROC of the univariate logistic model of FPG or HbA1c and the multivariate model of FPG and HbA1c were 0.771 (95% CI: 0.729–0.813), 0.796 (95% CI: 0.758–0.834), and 0.838 (95% CI: 0.804–0.871), respectively. All of these models had significantly higher AROCs than the KRS in predicting incident diabetes (Table 4).

**Table 4:** Comparison between the Korean Risk Score and risk prediction models of laboratory parameters.

	Korean Risk Score (KRS)	FPG model	HbA1c model	FPG and HbA1c model	Combined risk prediction model of KRS, FPG, and HbA1c (CRPM)
AROC(95% CI)	0.696 (0.655–0.737)	0.771 (0.729–0.813)	0.796 (0.758–0.834)	0.838 (0.804–0.871)	0.849 (0.818–0.880)
Change in AROC* (*P* value)		0.075 (0.011)	0.100 (<0.001)	0.142 (<0.001)	0.153 (<0.001)
NRI (%)* (*P* value)		27.3 (<0.001)	27.8 (<0.001)	45.2 (<0.001)	52.1 (<0.001)
IDI* (*P* value)		0.046 (<0.001)	0.058 (<0.001)	0.099 (<0.001)	0.105 (<0.001)
Change in AROC† (*P* value)					0.011 (0.016)
NRI (%)† (*P* value)					4.6 (0.264)
IDI† (*P* value)					0.006 (0.176)

Abbreviations: AROC, area under the curve of receiver operating characteristic curve; FPG, fasting plasma glucose; HbA1c, hemoglobin A1c; NRI, net reclassification index; IDI, integrative discrimination improvement.

*Changes in AROC, NRI, and IDI were calculated for each model compared to the KRS.

†Changes in AROC, NRI and IDI were calculated to compare FPG and HbA1c models and combined risk prediction models of KRS, FPG, and HbA1c.

To compare the risk classification, we calculated the improvement of the risk classification by the models of the laboratory parameters compared to the KRS by measuring the NRI and IDI (Table 4). The NRIs based on the risk categories of <5%, 5≤–<10%, 10≤–<15%, and ≥15% were 27.3% (95% CI: 13.9%-40.6%) for the FPG model, 27.8% (95% CI: 14.3%-41.3%) for the HbA1c model, and 45.2% (95% CI: 31.9%-58.5%) for the multivariate model of FPG and HbA1c. The addition of the KRS data to the CRPM increased the AROC (0.849, 95% CI: 0.818–0.880) by a small increment but did not improve the risk classification (NRI: 4.6%, 95% CI: -3.5% to 12.7%) (Table 4).

### Simulation of diabetes screening

To assess the performance of risk scores as a diabetes screening program and estimate the extent of misclassification, we simulated the application of the KRS and CRPM. First, we applied the KRS to the population without known diabetes at the baseline visit of the longitudinal study. Next, the CRPM was applied to the high-risk group classified by the KRS. Among 3,134 individuals without known diabetes at baseline, 1,513 individuals (48.3%) were predicted as having a high risk of undiagnosed diabetes after applying the KRS. Among those, the laboratory tests of FPG and HbA1c confirmed diabetes in 92 (6.1%). Among the remaining 1,421 individuals in the high-risk group, the CRPM classified 763 (53.7%) as having a high risk of incident diabetes. Among the 763, 90 (11.8%) developed diabetes. In the first step of screening, the KRS misclassified 13 out of 105 individuals (12.4%) with undiagnosed diabetes as low risk. In the second step of screening, the CRPM misclassified 11 out of 101 individuals (10.9%) with incident diabetes as low risk (S1 Fig).

## Discussion

In this external validation of diabetes risk scores in a study population of health screening program participants, the risk scores composed of non-laboratory parameters, including the KRS, demonstrated reasonable a performance for the screening of undiagnosed diabetes but were limited in their ability to predict incident diabetes. Comparing the KRS and laboratory parameters for the prediction of incident diabetes, the risk prediction model composed of FPG and HbA1c clearly showed a higher discrimination index, which was calculated as an AROC, and an improved risk classification over the KRS. The addition of the KRS to the risk prediction model of FPG and HbA1c increased the discrimination index, but only by a small increment and with no improvement in risk classification.

There is still no universal recommendation for the use of non-laboratory risk scores for diabetes screening. The ADA recommends diabetes screening with FPG, HbA1c, or oral glucose tolerance test (OGTT) for all adults over 45 years of age or younger than 45 years of age with a diabetic risk factor [14]. However, the National Institute for Health and Care Excellence (NICE) guidelines in the UK suggests the use of a validated risk-assessment tool prior to blood testing for adults aged 40 to 75 years and only offers blood testing to those who are classified as having a high risk of diabetes [32]. The Korean guideline for diabetes management recommends annual screening for diabetes with FPG, HbA1c, or OGTT for all adults over 40 years of age or over 30 years of age with a diabetic risk factor [33]. The Korean National Health Insurance Program provides a biennial health screening program, including fasting plasma glucose for all adults over the age of 40 [34]. These guidelines adopt considerably different strategies and should be evaluated for their performance and cost-effectiveness. Nonetheless, the KRS showed a reasonable performance and high negative predictive value both in current and previous studies [13]. The KRS was not the risk score with the highest discrimination index among the 17 scores validated in this study. Some risk scores showed better performances than the KRS despite being used in a different ethnicity and population from that for which they were developed. It was reported that risk scores developed in Western countries work poorly for Asian populations [35]. However, in this study, some risk scores developed in Western populations [16, 17, 22] showed higher discrimination indices than the Korean score. These results suggest that same ethnicity or nationality does not guarantee the generalizability of a risk score. External validation and recalibration of the risk scores should be performed before applying them to different populations, even with same ethnicity.

Intriguingly, both in the cross-sectional and longitudinal validation of diabetes risk scores, the AROCs of risk scores were higher in women than in men. Previous studies on diabetes risk scores also reported a higher AROC in women than in men [26, 29]. The reason of superior performance of diabetes risk scores for women was not evident. Factors that were not included in these risk scores might be responsible for the discrepant performance according to gender, so further studies are needed.

For the screening of undiagnosed diabetes, the risk scores of the non-laboratory parameters showed an acceptable performance. The negative predictive value was as high as 99%. This suggests that the low-risk group classified by the non-laboratory risk score has a very low possibility of having undiagnosed diabetes. The risk prediction model composed of basic laboratory parameters, including FPG and HbA1c, showed a superior performance for the prediction of incident diabetes than the non-laboratory models. However, testing FPG and HbA1c in the whole population as a mass-screening program has considerable costs. A two-step approach combining non-laboratory screening and laboratory screening can be a solution to this problem [12]. The non-laboratory risk score can be applied to the whole population for the screening of undiagnosed diabetes. Then, for those who have a high risk of undiagnosed diabetes, a simple blood test, including FPG and HbA1c, can detect or rule out diabetes. If the test results do not meet the criteria of diabetes, the predicted risk of incident diabetes can be calculated to determine who requires more intensive diabetes prevention programs and more frequent follow-up visits. This two-step approach can prevent unnecessary blood tests for low-risk individuals and reduce the cost of screening programs. Several previous studies also suggested a stepwise approach as an efficient diabetes screening program [12, 36]. The NICE guideline in the UK recommends a two-stage screening program [32]. In our simulation of the two-step approach, 13/105 (12.4%) of undiagnosed diabetes and 11/101 (10.9%) of incident diabetes were misclassified as low risk. This percentage is not negligible, but further adjustment of the cutoff values would reduce misclassification because the current cutoff values were determined by the highest Youden index without any clinical consideration.

Although the risk scores had high sensitivity and negative predictive value, they generally had low specificity, ranging from 42% to 72% for undiagnosed diabetes and 45% to 75% for incident diabetes. It should be emphasized that these risk scores are not confirmatory diagnostic tools. Instead, they should be considered as a screening tool for undiagnosed or future diabetes.

One of the limitations of this study is that the OGTT was not included in the definition of diabetes. Because FPG and HbA1c are less sensitive than the OGTT in diagnosing diabetes [37], the prevalence of undiagnosed diabetes could be underestimated. However, because the OGTT requires more time and cost than a fasting blood test, it is difficult to use for a mass screening program. The second limitation is that the risk prediction models of laboratory parameters were not externally validated. The development and validation of the models were not performed in independent cohorts. This could cause an overestimation of the prediction performance compared to the risk scores of the non-laboratory parameters that were externally validated in our cohort. The risk scores of non-laboratory parameters were not specifically developed for our data. We omitted or adjusted several parameters that were not identical to our data. This might lower the performance of non-laboratory risk scores. Another limitation is that the study population may not be a good representation of the general Korean population, because their participation in the health screening program can be affected by the individual’s socioeconomic status and health-seeking behavior. Lastly, due to the lack of information about the time of the diagnosis of incident diabetes, we could not apply the Cox regression model to our data.

In conclusion, the risk scores composed of non-laboratory parameters, including the KRS, can be useful self-assessment tools to estimate the risk of undiagnosed diabetes. However, their performance was inferior to the laboratory parameters for the prediction of incident diabetes.

## Supporting Information

S1 Fig Simulation of diabetes screening using the Korean Risk Score and the combined risk prediction model of the Korean Risk Score and laboratory parameters. This diagram summarizes the application of the Korean Risk Score (KRS) and combined risk prediction model of the KRS and laboratory parameters (CRPM) as a diabetes screening program. At first stage of screening, the KRS was applied to the study population of the longitudinal validation (total number of nondiabetic individuals was 3,134, which was 3,029 normal individuals plus 105 with undiagnosed diabetes at baseline). The KRS classified 1,513 individuals (48.3%) as at high risk for undiagnosed diabetes. The result of FPG and HbA1c confirmed 92 (6.1%) undiagnosed diabetes cases among them. At the second stage of screening, the CRPM was applied to the remaining 1,421 individuals (93.9%). The CRPM classified 763 individuals (53.7%) as at high risk for incident diabetes, and 90 (11.8%) among them developed diabetes at follow-up. ‘*’ denotes the total undiagnosed diabetes cases at baseline, which includes 92 (87.6%) confirmed cases and 13 (12.4%) missed cases. ‘**’ denotes the total future incident diabetes cases among the individuals who participated in the second stage of screening, which includes 90 (89.1%) predicted cases and 11 (10.9%) unpredicted diabetes cases.
(DOCX)

S1 Table Characteristics of the diabetes risk scores (DOCX)

S2 Table Area under the receiver operating characteristic curve of diabetes risk scores in men and women (DOCX)

S3 Table Univariate and multivariate logistic regression analysis of laboratory parameters for incident diabetes (DOCX)
