# Supplementary material for: Evaluation of Non-Laboratory and Laboratory Prediction Models for Current and Future Diabetes Mellitus: A Cross-Sectional and Retrospective Cohort Study
Source: PLoS One. 2016 May 23;11(5):e0156155. doi: 10.1371/journal.pone.0156155 (PMC4877115; doi:10.1371/journal.pone.0156155)

**Supplementary Data (S1 Fig)**

**Title: Evaluation of Non-laboratory and Laboratory Prediction Models for Current and Future Diabetes Mellitus: A Cross-Sectional and Retrospective Cohort Study**

**Short title:** Prediction of Current and Future Diabetes

**S1 Fig**. Simulation of diabetes screening using the Korean Risk Score and the combined risk prediction model of the Korean Risk Score and laboratory parameters

This diagram summarizes the application of the Korean Risk Score (KRS) and combined risk prediction model of the KRS and laboratory parameters (CRPM) as a diabetes screening program. At first stage of screening, the KRS was applied to the study population of the longitudinal validation (total number of non-diabetic individuals was 3,134 which was 3,029 normal individuals plus 105 undiagnosed diabetes at baseline). The KRS classified 1,513 (48.3%) individuals as high risk of undiagnosed diabetes. The result of FPG and HbA1c confirmed 92 (6.1%) undiagnosed diabetes among them. At second stage of screening the CRPM was applied to the remaining 1,421 (93.9%) individuals. The CRPM classified 763 (53.7%) individuals as high risk of incident diabetes and 90 (11.8%) among them developed diabetes at follow up. ‘*’ denotes the total undiagnosed diabetes at baseline, which is comprised of 92 (87.6%) confirmed and 13 (12.4%) missed cases. ‘**’ denotes the total future incident diabetes among the individuals who participated second stage of screening, which is comprised of 90 (89.1%) predicted and 11 (10.9%) unpredicted diabetes.


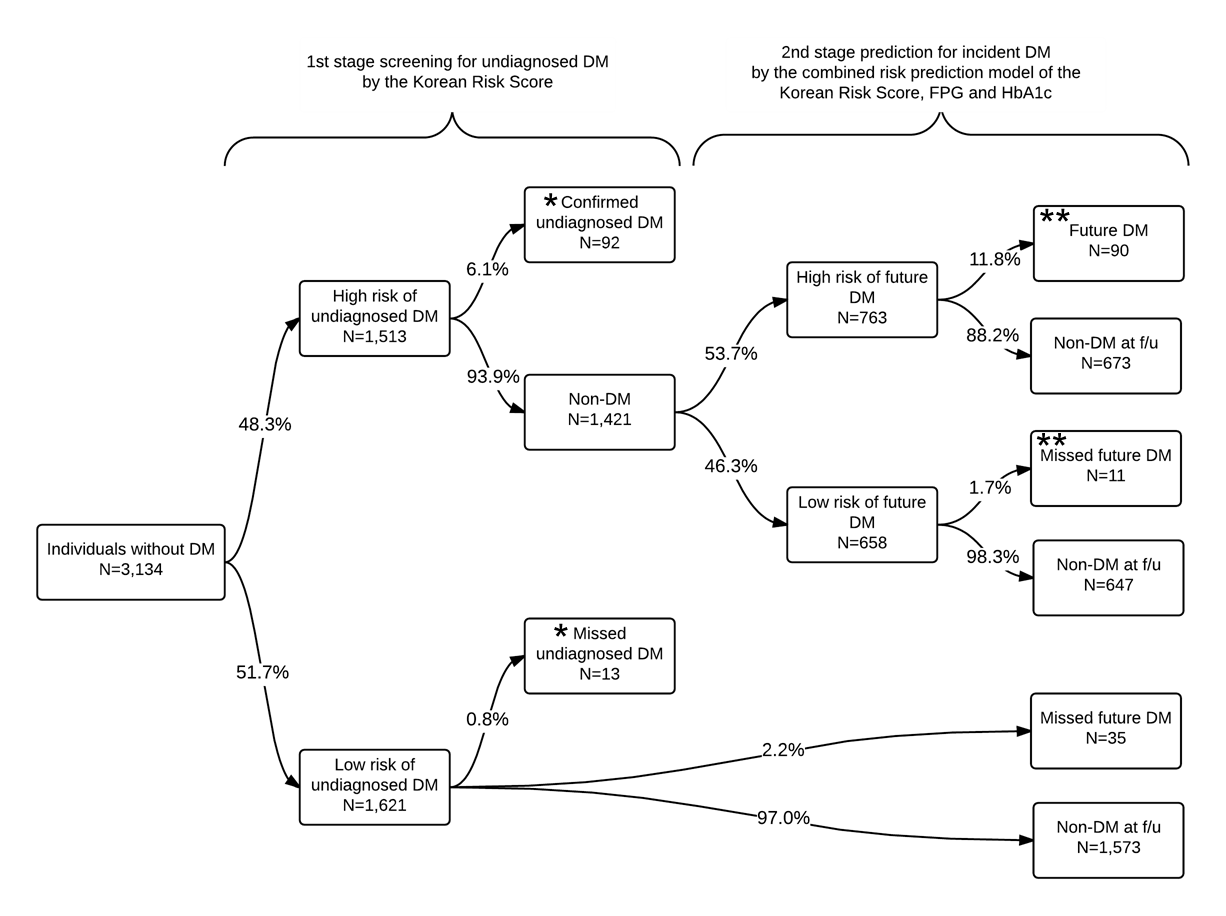

Supplement: S1 Fig — This diagram summarizes the application of the Korean Risk Score (KRS) and combined risk prediction model of the KRS and laboratory parameters (CRPM) as a diabetes screening program. At first stage of screening, the KRS was applied to the study population of the longitudinal validation (total number of nondiabetic individuals was 3,134, which was 3,029 normal individuals plus 105 with undiagnosed diabetes at baseline). The KRS classified 1,513 individuals (48.3%) as at high risk for undiagnosed diabetes. The result of FPG and HbA1c confirmed 92 (6.1%) undiagnosed diabetes cases among them. At the second stage of screening, the CRPM was applied to the remaining 1,421 individuals (93.9%). The CRPM classified 763 individuals (53.7%) as at high risk for incident diabetes, and 90 (11.8%) among them developed diabetes at follow-up. ‘*’ denotes the total undiagnosed diabetes cases at baseline, which includes 92 (87.6%) confirmed cases and 13 (12.4%) missed cases. ‘**’ denotes the total future incident diabetes cases among the individuals who participated in the second stage of screening, which includes 90 (89.1%) predicted cases and 11 (10.9%) unpredicted diabetes cases. (DOCX) [file pone.0156155.s001.docx]
