# Supplementary material for: Evaluation of Non-Laboratory and Laboratory Prediction Models for Current and Future Diabetes Mellitus: A Cross-Sectional and Retrospective Cohort Study
Source: PLoS One. 2016 May 23;11(5):e0156155. doi: 10.1371/journal.pone.0156155 (PMC4877115; doi:10.1371/journal.pone.0156155)
Supplement: S1 Table — (DOCX) [file pone.0156155.s002.docx]

**Supplementary Data (S1 Table)**

**Title: Evaluation of Non-laboratory and Laboratory Prediction Models for Current and Future Diabetes Mellitus: A Cross-Sectional and Retrospective Cohort Study**

**Short title:** Prediction of Current and Future Diabetes

**S1 Table.** Characteristics of the diabetes risk scores

|  |  |  |  |  |  |  |  | Development | | | | Validation | | |
| --- | --- | --- | --- | --- | --- | --- | --- | --- | --- | --- | --- | --- | --- | --- |
| Risk score | Author | Year | Nationality | Case/Total sample size | Longitudinal vs. cross-sectional | Follow-up duration | Risk predictors | AROC | Cut off | Sn | Sp | AROC | Sn | Sp |
| Korean Risk Score | Lee et al. | 2012 | Korea | 341/9602 | Cross-sectional |  | age, family history of diabetes, hypertension, waist circumference, smoking, alcohol | 0.73 | ≥5 | 81 | 54 | 0.729 | 80 | 53 |
| Australian score | Chen et al. | 2010 | Australia | 362/6060 | Longitudinal | 5 years | age, sex, ethinicity, family history of diabetes, history of high blood glucose, antihypertensive medications, smoking, physical inactivity, waist circumference, BMI | 0.783 | ≥12 | 74 | 68 | 0.66 |  |  |
| Danish score | Glumer et al. | 2004 | Denmark | 135/3250 | Cross-sectional |  | age, sex, BMI, Hypertension, physical activity at leisure time, parental history of diabetes | 0.804 | ≥31 | 73 | 74 | 0.803 | 76 | 72 |
| US screening score | Bang et al. | 2009 | USA | 147/5258 | Cross-sectional |  | age, sex, family history of diabetes, hypertension, obesity, physical activity | 0.83 | ≥5 | 79 | 67 | 0.74 | 72 | 62 |
| Japanese score (TOPICS-10 study) | Heianza et al. | 2013 | Japan | 965/33335 | Cross-sectional |  | age, sex, family history of diabetes, smoking, physical activity, BMI, hypertension | 0.771 | ≥8 | 73 | 68 | 0.806 | 74 | 71 |
| The Leiscester Risk Assessment score | Gray et al. | 2010 | UK | 206/6390 | Cross-sectional |  | age, sex, ethnicity, family history of diabetes, waist circumference, BMI, antihypertensive medication | 0.69 | ≥16 | 72 | 54 | 0.72 | 81 | 45 |
| Thai score | Aekplakorn et al. | 2006 | Thailand | 361/2667 | Longitudinal | 12 years | age, sex, BMI, waist circumference, hypertension, family history of diabetes | 0.74 | ≥7 | 77 | 60 | 0.75 | 84 | 53 |
| Finnish score | Alssema et al. | 2011 | Europe | 844/18301 | Longitudinal | 5 years | age, BMI, waist circumference, use of anti-hypertensives, history of gestional diabetes, sex, smoking, family history of diabetes | 0.764 | ≥7 | 76 | 63 |  |  |  |
| Brazilian score | Pires et al. | 2009 | Brazil | 118/1224 | Cross-sectional |  | age, BMI, hypertension | 0.772 | ≥18 | 76 | 67 | 0.72 | 86 | 45 |
| Indian score | Chaturvedi et al. | 2008 | India | 199/4044 | Cross-sectional |  | age, hypertension, waist circumference, family history of diabetes | 0.72 | ≥17 | 66 | 67 | 0.69 | 73 | 56 |
| Japanese score (Doi et al.) | Doi et al. | 2011 | Japan | 286/1935 | Longitudinal | 12 years | age, sex, family history of diabetes, waist circumference, BMI, hypertension, smoking, regular exercise | 0.700 | ≥14 | 63 | 67 | 0.691 |  |  |
| Chinese score | Gao et al. | 2010 | China | 194/1986 | Cross-sectional |  | age, sex, waist circumference, family history of diabetes | men: 0.635; women: 0.689 | men: ≥17; women: ≥14 | men: 64; women 81 | men: 57; women: 48 | men: 0.635; women: 0.689 |  |  |
| British score | Wannamethee et al. | 2011 | UK | 298/6848 | Longitudinal | 7 years | age, sex, family history, smoking, BMI, hypertension, waist circumference, pre-existing CHD | 0.764 | ≥6 | 79 | 62 |  |  |  |
| Rotterdam model | Baan et al. | 1999 | Netherlands | 118/1016 | Cross-sectional |  | age, sex, use of antihypertensives, physical inactivity, family history of diabetes, BMI |  |  |  |  | 0.74 | 55 | 72 |
| Oman score | Al-Lawati et al. | 2007 | Oman | 485/4881 | Cross-sectional |  | age, waist circumference, BMI, family history of diabetes, hypertension | 0.83 | >10 | 79 | 63 | 0.76 | 73 | 78 |
| French score (DESIR study) | Balkau et al. | 2008 | France | 203/3817 | Longitudinal | 9 years | men: waist circumference, smoking, hypertension; women: waist circumference, family history of diabetes, hypertension | men: 0.713; women: 0.827 |  |  |  |  |  |  |
| Kuwait score | Al Khalaf et al. | 2008 | Kuwait | 23/460 | Cross-sectional |  | age, waist circumference, hypertension, sibling history of diabetes | 0.82 | ≥32 | 87 | 64 |  |  |  |

Abbreviations: AROC, area under the curve of receiver operating characteristics curve; Sn, sensitivity; Sp, specificity; BMI, body mass index
