# Supplementary material for: Evaluation of Non-Laboratory and Laboratory Prediction Models for Current and Future Diabetes Mellitus: A Cross-Sectional and Retrospective Cohort Study
Source: PLoS One. 2016 May 23;11(5):e0156155. doi: 10.1371/journal.pone.0156155 (PMC4877115; doi:10.1371/journal.pone.0156155)
Supplement: S2 Table — (DOCX) [file pone.0156155.s003.docx]

**Supplementary Data (S2 Table)**

**Title: Evaluation of Non-laboratory and Laboratory Prediction Models for Current and Future Diabetes Mellitus: A Cross-Sectional and Retrospective Cohort Study**

**Short title:** Prediction of Current and Future Diabetes

**S2 Table.** Area under the curve of receiver operating characteristic curve of diabetes risk scores in men and women

|  | Cross-sectional validation | | | Longitudinal validation | | |
| --- | --- | --- | --- | --- | --- | --- |
|  | Men | Women | P value^a^ | Men | Women | P value^a^ |
| Korean Risk Score (KRS) | 0.698 (0.679-0.718) | 0.817 (0.795-0.840) | <0.001 | 0.650 (0.597-0.702) | 0.755 (0.689-0.822) | 0.015 |
| Australian score (AUSDRISK study) | 0.721 (0.702-0.740) | 0.844 (0.822-0.865) | <0.001 | 0.679 (0.622-0.736) | 0.768 (0.700-0.836) | 0.050 |
| Finnish score (DETECT-2 study) | 0.699 (0.678-0.719) | 0.825 (0.801-0.850) | <0.001 | 0.683 (0.629-0.737) | 0.761 (0.699-0.823) | 0.063 |
| Thai score | 0.706 (0.686-0.726) | 0.819 (0.795-0.843) | <0.001 | 0.673 (0.621-0.726) | 0.767 (0.710-0.824) | 0.018 |
| Danish score | 0.725 (0.707-0.744) | 0.820 (0.795-0.845) | <0.001 | 0.672 (0.619-0.726) | 0.720 (0.649-0.791) | 0.295 |
| The Leiscester Risk Assessment score | 0.718 (0.699-0.737) | 0.817 (0.790-0.844) | <0.001 | 0.666 (0.611-0.722) | 0.718 (0.649-0.788) | 0.253 |
| Japanese score (TOPICS-10 study) | 0.721 (0.702-0.740) | 0.831 (0.808-0.855) | <0.001 | 0.677 (0.623-0.732) | 0.735 (0.673-0.798) | 0.169 |
| Chinese score | 0.707 (0.688-0.726) | 0.812 (0.788-0.836) | <0.001 | 0.676 (0.625-0.727) | 0.749 (0.691-0.808) | 0.063 |
| Indian score | 0.698 (0.678-0.717) | 0.815 (0.791-0.838) | <0.001 | 0.650 (0.596-0.704) | 0.741 (0.679-0.804) | 0.031 |
| Japanese score (Doi et al.) | 0.705 (0.684-0.725) | 0.767 (0.734-0.801) | 0.002 | 0.651 (0.597-0.705) | 0.702 (0.611-0.793) | 0.345 |
| ADA questionnaire | 0.722 (0.703-0.740) | 0.818 (0.792-0.844) | <0.001 | 0.664 (0.610-0.718) | 0.695 (0.617-0.772) | 0.523 |
| Brazilian score | 0.698 (0.679-0.717) | 0.816 (0.791-0.840) | <0.001 | 0.663 (0.608-0.717) | 0.699 (0.624-0.774) | 0.441 |
| Oman score | 0.697 (0.677-0.717) | 0.786 (0.762-0.811) | <0.001 | 0.670 (0.618-0.722) | 0.701 (0.628-0.773) | 0.496 |
| British score | 0.673 (0.652-0.694) | 0.759 (0.726-0.792) | <0.001 | 0.622 (0.567-0.677) | 0.702 (0.620-0.783) | 0.112 |
| French score (DESIR study) | 0.657 (0.636-0.677) | 0.804 (0.780-0.828) | <0.001 | 0.618 (0.560-0.675) | 0.742 (0.677-0.807) | 0.005 |
| Rotterdam model | 0.676 (0.655-0.698) | 0.760 (0.732-0.788) | <0.001 | 0.609 (0.553-0.665) | 0.640 (0.548-0.732) | 0.573 |
| Kuwait score | 0.669 (0.649-0.689) | 0.724 (0.694-0.755) | 0.003 | 0.618 (0.566-0.670) | 0.631 (0.551-0.710) | 0.797 |

^a^P value for the comparison between ROC curves for men and women was calculated using DeLong’s method.
