# Supplementary material for: Evaluation of Non-Laboratory and Laboratory Prediction Models for Current and Future Diabetes Mellitus: A Cross-Sectional and Retrospective Cohort Study
Source: PLoS One. 2016 May 23;11(5):e0156155. doi: 10.1371/journal.pone.0156155 (PMC4877115; doi:10.1371/journal.pone.0156155)
Supplement: S3 Table — (DOCX) [file pone.0156155.s004.docx]

**Supplementary Data (S3 Table)**

**Title: Evaluation of Non-laboratory and Laboratory Prediction Models for Current and Future Diabetes Mellitus: A Cross-Sectional and Retrospective Cohort Study**

**Short title:** Prediction of Current and Future Diabetes

**S3 Table**. Univariate and multivariate logistic regression analysis of laboratory parameters for incident diabetes

| Laboratory parameters | Univariate analysis | | Multivariate analysis | | |
| --- | --- | --- | --- | --- | --- |
|  | Odds ratio (95% CI) | P value | β coefficient | Odds ratio (95% CI) | P value |
| FPG | 1.11 (1.09-1.13) | <0.001 | 0.074 | 1.077 (1.056-1.097) | <0.001 |
| HbA1c | 37.406 (20.767-67.377) | <0.001 | 2.876 | 17.738 (9.408-33.443) | <0.001 |
| Total cholesterol | 1.008 (1.003-1.013) | <0.001 |  |  |  |
| HDL cholesterol | 0.971 (0.957-0.985) | <0.001 |  |  |  |
| Triglyceride | 1.005 (1.003-1.006) | <0.001 |  |  |  |

Abbreviations: FPG, fasting plasma glucose; HbA1c, hemoglobin A1c
